# Vitamin D levels in schoolchildren: a cross-sectional study in Kuwait

**DOI:** 10.1186/s12887-017-0963-0

**Published:** 2017-12-22

**Authors:** Khulood Othman Alyahya

**Affiliations:** Science Department, College of Basic Education, Public Authority for Applied Education and Training (PAAET), Ardhyia, Kuwait

**Keywords:** Vitamin D, 25(Oh)D, Children, Age, PTH, Kuwait

## Abstract

**Background:**

Ongoing studies in the Middle East, particularly in the Arabian Gulf countries, have reported extremely low levels of serum vitamin D across age and gender. In Kuwait, vitamin D deficiency is prevalent in adolescent girls and in adult women. A number of risk factors have been reported, among which gender, age, and obesity are a few. Because adequate vitamin D status is necessary to promote bone mineral accrual in childhood, and because low vitamin D levels have been associated with a wide range of health problems, there is concern that growing children with low vitamin D may be at higher risk for developing diseases. The aim of this study was to assess vitamin D levels in elementary schoolchildren.

**Methods:**

Kuwaiti schoolchildren were recruited and assessed for their serum vitamin D, 25(OH)D, parathyroid hormone (PTH) and adjusted serum calcium (adj-Ca). Anthropometric measurements and data on lifestyle and health status were recorded during an interview.

**Results:**

In a total of 199 schoolchildren, median (IQR) age was 8.5 (7.0–9.5 years), 25(OH)D was 30 (22–39 nmol/L), PTH was 4.7 (3.8–5.9 pmol/L), and adj-Ca was 2.39 (2.33–2.44 mmol/L). Boys had higher levels of 25(OH)D (18.3% vs 6.6% had levels ≥50 nmol/L) and lower levels of PTH (94.6% vs 80.2% had levels <7 pmol/L) than girls. Significant risk factors for 25(OH)D levels <25 nmol/L included being ≤8.5 years old (OR 4.95, 95% CI: 1.92–12.74), having PTH ≥7 pmol/L (OR 2.28, 95% CI: 1.17–4.46), being female (OR 2.44, 95% CI: 1.22–4.88), and being overweight or obese (OR 2.18, 95% CI: 1.11–4.26).

**Conclusions:**

The results show relatively low levels of 25(OH)D in young schoolchildren in Kuwait, with lower levels in girls. Given the association of 25(OH)D with a wide range of ailments, it is necessary to further examine the causes and risk factors of low vitamin D in this age group to prevent associated health problems.

## Background

Studies in the Middle East have repeatedly confirmed that there are low serum levels of vitamin D across regions, ages, and genders [1], with further lower levels among populations in Arabian Gulf countries [2, 3]. The high temperature, traditional clothing, and availability of private transportation for most of the population have most likely discouraged regular exposure to the sun.

Children, among other age groups, have demonstrated a moderate to high prevalence of vitamin D deficiency in the Middle East. In a sample of 331 Saudi children in Riyadh aged 6–17 years, vitamin D levels <50 nmol/L were found in 71.6% of the children [4]. Similarly, 78.8% of 293 adolescent girls (11–18 years) in UAE were found to be deficient with levels <27.5 nmol/L [5]. In Qatar, a study showed that serum 25(OH)D levels <75 nmol/L were prevalent among 61% of 11–166-year-old Qatari adolescents, 29% of 5–10 year old children, and 9.5% of children below 5-years old, among whom delayed milestones, fractures, rickets, and gastroenteritis were more common [6]. Vitamin D deficiency in children is also common in other non-Gulf countries. The prevalence of vitamin D deficiency in Lebanese schoolchildren (aged 13.3 ± 1.6 years) was reported to be 52% [7], and in a similar study in Iranian schoolchildren in Tehran, 29% of boys and 66.6% of girls had levels of serum vitamin D < 25 nmol/L [8]. Levels ≥50 nmol/L were found in only 32.2% boys and 15.1% girls.

Adolescent females in Kuwait have been assessed before. A total of 233 females aged 10–18 years were found to have serum vitamin D levels <75 nmol/L in both winter and summer, among which 78.4% had levels <25 nmol/L [9]. Earlier studies in younger children in Kuwait have been carried out, but with a special focus on the prevalence and symptoms of rickets and vitamin D rickets as a result of vitamin D deficiency [10–12].

Some of the well-known risk factors for vitamin D deficiency are female gender, certain age groups such as neonates, preschool children, and the elderly, obesity, veiling, dark skin, African and Asian ethnicity, winter, and low socio-economic status [2].

It is well-understood that adequate vitamin D status is important for optimal bone health and that low levels of vitamin D are associated with elevated levels of parathyroid hormone and accordingly inadequate bone mineralization [13]. More recently, vitamin D deficiency has been linked to a wide range of ailments and diseases [14]. Adults with low levels of vitamin D have been shown to have higher risk for heart disease, diabetes, cancers, high blood pressure and immune-related diseases [14, 15]. Children with diabetes, metabolic syndrome, asthma, dermatitis, and anemia have also been found to have lower levels of vitamin D compared to controls [16–19].

There is evidence to support the beneficial role of vitamin D in a number of non-skeletal tissues and systems. For example, there is some evidence of the role of vitamin D in cardiovascular diseases. Experimental animal and cell culture studies have demonstrated a variety of effects by which vitamin D receptor activation protects the cardiovascular system [14].

There is also evidence to show that vitamin D has a role in increasing the effect of innate immune processes while restraining the adaptive immune system, leading to improved outcomes in autoimmune diseases. In addition, vitamin D has been found to have an antimicrobial effect in certain bacterial infections, such as those with frequent respiratory tract infections and dental caries [14]. Studies have found an inverse correlation between inflammatory markers such as TNF-α and C-reactive protein and 25(OH)D levels, which suggests that inflammation is a common factor between most non-skeletal health disorders and low 25(OH)D levels [15].

International health organizations such as the Institute of Medicine, IOM, have set specific guidelines and recommendations to overcome the widespread vitamin D deficiency. They have raised awareness of its importance in promoting and maintaining overall health. They have set guidelines to encourage raising serum levels of vitamin D via safe exposure to sun rays and by consuming more vitamin D rich foods and beverages [20]. The US Department of Agriculture’s Nutrient Database lists foods and their vitamin D levels on their website for general use. On the other hand, food companies have been encouraged to fortify more foods for consumers based on scientific evidence that consumption of fortified milk for 24 months significantly increased 25(OH)D levels and reduced bone loss in the lumbar spine and hip [21]. Supplementation is recommended when attempts to obtain enough dietary vitamin D or sun exposure fail. The IOM recommends that infants up to one-year old require 400 IU, and children 1–18 years old should receive 600 IU [22].

In addition, various organizations and institutions have raised the cutoff for normal serum vitamin D levels that was once as low as 25 nmol/L to levels that are suggested to promote overall health. Despite the fact that the new cutoff varies between institutions, there is a general consensus that optimal levels of 25(OH)D should be above 50 nmol/L [20]. This cutoff is based on evidence that lower levels have been associated with various disease outcomes, suggesting that higher levels may be protective [14].

There is also a general agreement that screening high risk individuals for vitamin D deficiency is required. Individuals at risk include but are not limited to chronically ill and hospitalized patients and those with osteoporosis, renal or liver diseases, pregnant and lactating women, infants and the elderly [23]. Children at high risk require no less than 1000 IU of dietary vitamin D [22].

Thus far, these international guidelines have been adapted in various populations, including the Arabian Gulf region. However, just recently, the Prince Mutaib Chair for Biomarkers of Osteoporosis PMCO in King Saud University, Riyadh, KSA, in cooperation with the European Society for Clinical and Economic Aspects of Osteoporosis and Osteoarthritis, have formulated unified guidelines for the prevention, diagnosis and treatment of vitamin D deficiency in the Saudi population [24]. The guidelines take into account the ethnic, geographical and cultural factors that exist in the region, which are also major determinants of vitamin D status. The PMCO, therefore, recommends vitamin D supplementation for all of those whose serum vitamin D levels fall below 50 nmol/L (the deficiency cutoff), while frail, osteoporotic and older patients should target a level of 75 nmol/L.

With regard to children, supplementation is recommended for children of all ages, especially females as follows: infants up to 6-months old should receive 400 IU/day, infants 6–12-months old should receive 400–600 IU/day, children over one-year old should receive 600–1000 IU, and obese children should receive 1200–2000 IU/day [24]. Supplementation of 1000–2000 IU/day is also recommended during pregnancy. Similarly, the UAE has set some guidelines to overcome the widespread vitamin D deficiency in the region, through which supplementation is recommended for everyone [25]. Both Saudi and UAE guidelines are perhaps more suitable for the rest of the Arabian Gulf populations.

The aim of this study was to assess serum vitamin D status in young schoolchildren, and to determine the risk factors associated with low levels of vitamin D.

## Methods

This cross-sectional study was conducted in spring and early summer 2011 (from April to the end of May). The study was approved by the Joint Committee for the Protection of Human Subjects in Research combined by Kuwait Institute for Medical Specialization KIMS - Ministry of Health and AbdulMihsin Al-Abdulrezzag Health Sciences Centre HSC - Health Sciences Centre - Kuwait University.

Another approval was sought and granted by the Ministry of Health and Head of the primary health care centers to recruit and examine the participating students. Accordingly, three primary health care centers were included: Qurtuba, Yarmook and Al-Khaldyia.

A third approval was required and granted by the Centre for Educational Research, Ministry of Education to allow entry into public elementary schools. This approval was necessary to obtain a convenience sample of young schoolchildren 5–11 years old.

Invitation letters were distributed in a number of schools in all governorates and were sent with the students to take home and show their parents. The parents of students who were interested in participating in the study returned the invitation letters with their signature and contact numbers to further set an appointment. The returned invitation letters were screened to exclude those with diseases that might interfere with vitamin D synthesis, including skin, liver, kidney and gastrointestinal diseases, those taking certain medication or vitamin/mineral supplements, post-pubertal students, and non-Kuwaitis. Accordingly, the eligible students were contacted later to set an afternoon appointment at one of the health care centers.

When the students, accompanied by their parents, were presented for examination, the study and methods were explained in further detail. Consent and assent forms were signed before the examination occurred. Consent forms were signed by the parent, while assent forms were signed by ≥7 years old students.

Three trained dieticians completed Interviewer-administered questionnaires and body measurements in 20–30 min. Almost all students were accompanied by their mothers for clarity in answering questions. Multiple questionnaires were used to collect data on socioeconomic status, lifestyle, physical activity and sun exposure.

Height, weight, head and wrist circumference were measured, and body mass index (BMI) obesity index was calculated based on the international cut-off for body mass index for overweight and obesity by sex between 2 and 18 years old [26]. A normal BMI is <85th percentile, overweight is 85-95th percentile, and obesity is >95th percentile.

Skin color/type was evaluated based on the Fitzpatrick classification of skin phototype [27], which classifies the skin according to its susceptibility to sunburn and tanning into 6 types. Skin color/types were regrouped into light (2, 3), medium (4), and dark (5, 6). Skin type 1 was not found among the children. Sun exposure was evaluated by asking the parents to provide a rough estimate of the average daily time their child spent under the sun in the past month. The parent responded by choosing up to 5 min, up to 15 min, or 30 min/or more.

Physical activity was evaluated by asking the parent to provide a rough estimate of their child’s level of physical activity by engaging in active playing, running, jumping, climbing, playing football or basketball, or other activities. The parent responded by choosing daily, weekly, or rarely.

Blood was withdrawn by a nurse/lab technician and was sent to, London, UK for measurement of serum vitamin D, 25(OH)D, parathyroid hormone (PTH), adjusted serum calcium (adj-Ca). Elecsys Vitamin D total assay using an immunoassay analyzer (Roche diagnostics, USA) was used to quantify 25(OH)D, with inter- and intra-assay CV%s of 3.43% and 5.44%, respectively. Intact PTH was measured with an electrochemiluminescence immunoassay using an immunoassay analyzer (Roche, USA) with inter- and intra-assay CV%s of 1.6% and 3.9%, respectively, and adj-Ca was measured with Calcium Gen.2 using Cobas c analyzer (Roche diagnostics, USA) with inter- and intra-assay CV%s of 0.7% and 0.9%, respectively.

Statistical analysis was performed using SPSS, version 22. Normality was assessed using the Kolmogorov-Smirnov test. The majority of variables were not normally distributed, and non-parametric tests were used. Median and IQR were used to report continuous variables. Spearman’s correlation tests, Mann-Whitney U tests, and Kruskal-Wallis H tests were used to assess associations with 25(OH)D. Both the M-W and K-W tests compare mean ranks of subgroups instead of their medians, and are thus listed, along with the *p-value*, in Table 4. Different from the mean and median, a mean rank represents the mean of rank scores of a subgroup after ranking the values of all subgroups of an independent variable in a single order [28]. A high mean rank corresponds to a high value. A significant difference between mean ranks across subgroups is indicated by a *p-value ≤ 0.05*.

Logistic regression was performed to identify the risk factors for vitamin D deficiency. Potential risk factors were initially assessed for their associations with 25(OH)D using non-parametric tests. Those that were significantly associated with 25(OH)D were included as independent variables in the binary regression model. The dependent variable was vitamin D deficiency, which was set at 25 nmol/L of 25(OH)D. Values below 25 nmol/L were considered as deficient. While its generally agreed on that 50 nmol/L marks sufficiency in 25(OH)D levels [22], there is also some agreement that 25 nmol/L serves as cut off for deficiency [22]. Levels between 25 and 50 nmol/L are considered insufficient. In this data set, more cases fell below 25 nmol/L compared to 50 nmol/L. Thus, using 25 nmol/L cut off point is more likely to represent a better statistical outcome.

## Results

### Characteristics of the schoolchildren

In a total of 199 Kuwaiti schoolchildren, the median age was 8.5 years (IQR; 7.0–9.5), and median BMI was 17.7 (IQR; 15.6–21.0). No differences were found between boys and girls in age or BMI (Table 1). The highest percentage of participants was from the Asma governorate (22.6%), and the lowest was from Jahara (12.6%) (Table 2). The oldest age group in the study was 8.5–9.49 years (26%), and the lowest was 5.5–6.49 years (5%). Further, 24.6% were found to be overweight, and 18.6% were obese. Obesity was more prevalent in boys (23.7%) than in girls (14.2%) (Table 2). The most common skin color was the medium skin-tone (58.8%), followed by the light skin-tone (32.7%). However, more boys had a darker skin-tone (11.8%) compared to girls (5.7%). Sun exposure up to 30 min occurred in only 30.2% of the schoolchildren and included more boys than girls (38.7% vs. 22.6%). In addition, daily physical activity did not occur in more than 52.8% of the schoolchildren. Fewer boys (12.9%) than girls (27.4%) were physically inactive.

**Table 1:** Anthropometric mesurements of the schoolchildren

Measurement	Total (*n* = 199)				Boys (*n* = 93)				Girls (*n* = 106)				*p-value* ^*^
	Median	IQR	Min	Max	Median	IQR	Min	Max	Median	IQR	Min	Max	
Age years	8.5	7.0–9.5	5.5	11.0	8.0	7.0–9.0	5.5	11.0	8.5	7.0–9.5	5.5	11.0	*0.267*
Weight kg	29.0	24.0–36.2	14.0	80.5	28.5	24.1–38.8	16.5	80.5	29.2	23.0–35.6	14.0	71.0	*0.688*
Height cm	128.0	121.0–135.0	29.3	158.5	128.0	120.3–133.0	29.3	153.0	128.5	121.9–135.1	99.0	158.5	*0.326*
BMI kg/cm2	17.7	15.6–21.0	12.7	36.3	18.1	15.9–21.7	12.9	36.3	17.7	15.4–19.8	12.7	31.7	*0.212*
Head circumference cm	53.0	52.0–54.0	35.0	58.0	53.0	52.0–54.0	35.0	58.0	53.0	51.0–54.0	49.0	58.0	*0.108*
Wrist cm	14.0	13.0–15.0	11.0	40.0	14.0	13.0–15.0	11.0	40.0	13.8	13.0–14.5	11.0	17.0	*0.001*

^*^ *P-value* indicates differences between boys and girls using Mann-Whitney U test

**Table 2:** Characteristics of the schoolchildren

Variable	Catagories	Total (*n* = 199)		Boys (*n* = 93)		Girls (*n* = 106)	
		n	%	n	%	n	%
Governorate	Asma	45	*22.6*	17	*18.3*	28	*26.4*
	Hawalli	26	*13.1*	10	*10.8*	16	*15.1*
	Farwania	29	*14.6*	19	*20.4*	10	*9.4*
	Mubarak Al-Kabir	41	*20.6*	27	*29.0*	14	*13.2*
	Ahmedi	33	*16.6*	9	*9.7*	24	*22.6*
	Jahara	25	*12.6*	11	*11.8*	14	*13.2*
Age years	5.5–6.49	10	*5.0*	5	*5.4*	5	*4.7*
	6.5–7.49	41	*20.6*	19	*20.4*	22	*20.8*
	7.5–8.49	45	*22.6*	25	*26.9*	20	*18.9*
	8.5–9.49	52	*26.1*	25	*26.9*	27	*25.5*
	9.5–10.49	38	*19.1*	13	*14.0*	25	*23.6*
	10.5–11.0	13	*6.5*	6	*6.5*	7	*6.6*
Obesity index	non	113.0	*56.8*	49.0	*52.7*	64.0	*60.4*
	overweight	49.0	*24.6*	22.0	*23.7*	27.0	*25.5*
	obese	37.0	*18.6*	22.0	*23.7*	15.0	*14.2*
Skin color	light	65	*32.7*	25	*26.9*	40	*37.7*
	medium	117	*58.8*	57	*61.3*	60	*56.6*
	dark	17	*8.5*	11	*11.8*	6	*5.7*
Sun exposure	up to 5 min	99	*49.70*	41	*44.1*	58	*54.7*
	up to 15 min	40	*20.10*	16	*17.2*	24	*22.6*
	up to 30 min	60	*30.20*	36	*38.7*	24	*22.6*
Physical activity	rarely	41	*20.60*	12	*12.9*	29	*27.4*
	weekly	53	*26.60*	27	*26.6*	26	*24.5*
	daily	105	*52.80*	54	*58.1*	51	*48.1*

### 25(OH)D and associations

The median 25(OH)D level was 30 nmol/L (IQR; 22–39), PTH was 4.7 pmol/L (IQR; 3.8–5.9), and adj-Ca was 2.39 mmol/L (IQR; 2.33–2.44) (Table 3). A significant difference was found between boys and girls in 25(OH)D (*p = 0.0001*) and PTH (*p = 0.001*) but not in serum calcium, both adjusted and unadjusted (*p > 0.05*). Boys had a higher median 25(OH)D level than girls (34.0 vs. 27.0 nmol/L) and a lower PTH (4.2 vs. 5.2 pmol/L).

**Table 3:** Clinical mesurements of the schoolchildren

Measurement*	Total (*n* = 199)				Boys (*n* = 93)				Girls (*n* = 106)				*P-value*
	Median	IQR	Min	Max	Median	IQR	Min	Max	Median	IQR	Min	Max	
25(OH)D nmol/L	30.0	22.0–39.0	5.0	89.0	34.0	27.0–47.0	12.0	89.0	27.0	18.0–35.0	5.0	71.0	***0.001***
PTH pmol/L	4.70	3.80–5.90	1.90	20.70	4.20	3.55–5.40	1.90	9.00	5.20	4.08–6.33	2.00	20.70	***0.001***
Calcium mmol/L	2.47	2.39–2.52	1.43	2.71	2.45	2.36–2.53	2.09	2.70	2.47	2.40–2.52	1.43	2.71	*0.245*
Adj Calcium mmol/L	2.39	2.33–2.44	2.14	2.59	2.39	2.33–2.45	2.14	2.58	2.39	2.34–2.44	2.17	2.59	*0.749*

*25(OH)D serum hydroxyvitamin D, *PTH* parathyroid hormone, *Calcium* unadjusted serum calcium, *Adj Calcium* adjusted serum calcium

**Bold** ***p-value*** indicates significant differences between boys and girls

Spearman’s correlations showed a significant correlation between 25(OH)D and PTH (*r* ^*2*^ *= −0.367, p = 0.0001*), age (*r* ^*2*^ *= −0.224, p = 0.001*) and BMI (*r* ^*2*^ *= −0.229, p = 0.001*), whereas no correlation was found with serum calcium (*p > 0.05*). There were significant differences in 25(OH)D among different obesity indexes in girls (*p = 0.044*) but not in boys (*p = 0.214*) (Table 4). There were also significant differences in 25(OH)D among different skin-color types in girls (*p = 0.01*). There was no difference in 25(OH)D across the different physical activity levels or sun exposure times (*p > 0.05*), nor was there any significant differences across the six governorates of Kuwait (*p > 0.05*).

**Table 4:** Differences in 25(OH)D between subgroups by their mean ranks

Variable	Catagories	Total (*n* = 199)		Boys (*n* = 93)		Girls (*n* = 106)	
		Mean rank	*p-value*	Mean rank	*p-value*	Mean rank	*p-value*
Governorate			*0.559*		*0.300*		*0.953*
	Asma	97.13		51.68		50.18	
	Hawalli	102.63		53.10		55.38	
	Farwania	93.76		35.03		56.80	
	Mubarak Al-Kabir	114.30		50.06		57.75	
	Ahmedi	90.59		53.72		50.58	
	Jahara	98.62		41.91		56.39	
Age years			**0.016**		**0.045**		**0.025**
	5.5–6.49	136.65		73.50		67.60	
	6.5–7.49	110.39		39.42		71.57	
	7.5–8.49	112.98		55.60		52.83	
	8.5–9.49	89.25		45.44		42.96	
	9.5–10.49	80.57		35.58		48.54	
	10.5–11.0	93.92		44.33		46.93	
Obesity index			*0.084*		*0.214*		**0.044**
	non	107.85		51.47		59.10	
	overweight	91.41		44.02		48.28	
	obese	87.42		40.02		39.00	
Skin color			*0.505*		*0.936*		**0.010**
	light	105.65		45.34		62.20	
	medium	98.51		47.68		50.62	
	dark	88.65		47.23		24.33	
Sun exposure			*0.618*		*0.465*		*0.663*
	up to 5 min	97.38		49.04		51.40	
	up to 15 min	97.34		39.47		58.13	
	up to 30 min	106.09		48.03		53.96	
Physical activity			*0.650*		*0.170*		*0.922*
	rarely	90.20		39.88		57.41	
	weekly	110.85		54.87		54.90	
	daily	98.35		44.65		52.26	

*Bold P-value* indicates significant difference in 25(OH)D between subgroups using Kruskall-Wallis H test

### Significant risk factors for deficient 25(OH)D

In a binary logistic regression analysis, risk factors for low 25(OH)D (level < 25 nmol/L) were being 8.5 years old or older, having a PTH level of ≥7 pmol/L, being a female and being overweight or obese (Table 5). The model met the Hosmer & Lemeshow Goodness of Fit test (*p = 0.9* i.e.*, >0.05*). These significant risk factors accounted for 16–22% (*R* ^*2*^) of the variation in 25(OH)D in almost 75% of the cases (percentage accuracy). The strongest predictor, however, was age (OR; 4.95, 95% CI: 1.92–12.74), meaning that children aged 8.5 years and older were almost five times more likely to be deficient (<25 nmol/L) compared to younger children in this study.

**Table 5:** Risk factors of low vitamin D levels (<25 nmol/L) in schoolchildren

Independent variables^a^	*Odds ratio*	95% C.I.		*P-value*
		Lower	Upper	
Age (<8.5 yrs. vs. older)	*4.954*	1.926	12.742	*.001*
Gender (male vs. female)	*2.444*	1.225	4.877	*.011*
PTH (<7 pmol/L vs. higher)	*2.282*	1.166	4.464	*.016*
Overweight (& obese vs. normal)	*2.175*	1.110	4.262	*.024*

^a^Entered in logistic regression, with goodnes of fitness >0.05, an *R squared value of 0.159–0.223* and a percentage accuray of 75% overall

## Discussion

### Vitamin D associations with lifestyle in children

The current sample was a convenience sample of Kuwaiti schoolchildren 5–11 years old, with similar numbers of students recruited from each governorate and each gender. In some studies, residence and socio-economic status have been shown to have a significant effect on 25(OH)D levels [7], while others found no effect [8]. Because all governorates of Kuwait enjoy a relatively similar degree of services and prosperity, the location of residence should have a trivial impact on 25(OH)D levels.

With regard to physical activity, only 52.8% of the children were physically active on a daily basis. However, no difference in 25(OH)D was found between those who were more or less active. This finding is not congruent with the literature. A study by Al-Othman et al. [4] had found that 25(OH)D was significantly lower in a physically inactive group compared to an active group (17.7 + 1.6 nmol/L vs 22.7 + 1.5 nmol/L, *p < 0.05*). The inactive group also had a higher fasting glucose level and a higher BMI (*p < 0.05*), which have also been shown to correlate with 25(OH)D [16, 29]. The study also found that 25(OH)D levels were significantly lower in the group that reported no sun exposure and that the levels increased with increasing time under the sun. In our schoolchildren, spending up to 5 min in the sun was generally common (49.7%) despite the beautiful weather during which recruitment was carried out (April and May). More outdoor time and sun exposure were expected given the spring season. Clearly, more sun exposure and daily physical activity should be encouraged among these young children as part of a holistic, healthier lifestyle.

Skin pigmentation impacts the rate of endogenous vitamin D production in the skin. Darker skin requires more time in the sun to produce the same amount of vitamin D as lighter skin. Studies have shown that skin color/type may contribute to the variation in 25(OH)D, but results are inconsistent. Skin color, in the current study, had a linear relation with 25(OH)D, but the relation was only significant in girls (*p = 0.01*). Girls with lighter skin had the highest mean rank of 25(OH)D, whereas girls with the darkest skin had the lowest mean rank.

Other lifestyle-related predictors of 25(OH)D levels include season, nutrition and dress type [2]. However, these were not investigated in the present study.

### Vitamin D and common risk factors in children

The risk factors in this study were similar to what has been reported in Western populations [2]. Age, gender, PTH and obesity appear to be global risk factors for low 25(OH)D.

#### PTH levels

The inverse relation between 25(OH)D and PTH indicates their interaction in constructing and preserving bone tissue. This finding has been elaborated extensively in the literature [30–32].

Elevated PTH levels are associated with elevated bone turnover markers, suggesting that PTH stimulates bone resorption. Thus, it is important that vitamin D levels remain available to prevent bone resorption. Studies have shown that bone mass and mineralization during childhood and adolescence is critical across the whole lifespan [33].

In the current study, 25(OH)D levels were inversely associated with PTH levels (*R* ^*2*^ *= −0.373, p < 0.005*). However, with regard to gender, only girls had a significant relation. This finding may be explained by the positive impact of estrogen on PTH levels [34].

#### Gender

Being a female was a risk factor for low 25(OH)D and elevated PTH levels. This finding has been documented extensively in the literature. Compared to males, females of all ages are more susceptible to low vitamin D status. Differences between genders in Middle Eastern studies have been thought to be a result of the concealing clothing and reduced outdoor activity, which reduces sun exposure. However, the consistency of this association throughout age groups suggests that orchestrating factors might be physiological rather than cultural. This gender-dependent relation suggests a strong regulation by sex hormones, particularly estradiol and estrogens, in the synthesis and metabolism of vitamin D [35, 36].

#### Age

Age and 25(OH)D are significantly correlated in the majority of studies. Studies in the Middle East have shown that 25(OH)D increases with age in adult females and males, while it decreases with age in children and adolescence [4, 5, 8, 9, 31, 37]. This trend is interesting because it is contrary to the general findings in the Western populations where the opposite happens. The physiological explanation for the decrease in 25(OH)D in older adults is due to a significant decline in the skin’s capacity to produce vitamin D [38].

In our study, 25(OH)D was inversely correlated with age (*R* ^*2*^ *= −0.265, p < 0.005*), and the same was found previously in adolescent girls (10–18 years) in Kuwait [9]. Older adolescent girls had lower 25(OH)D values compared to younger girls. Puberty is a critical time during which increased demand on vitamin D and minerals occurs to form bone, which is also governed by increased sex hormones [39]. Ultimately, this process can result in reduced circulating levels of 25(OH)D, especially if the supply is limited [38]. An interesting finding was that 25(OH)D levels were shown to be inversely related with gain in bone area, bone mass content, and bone mass density (*p < 0.05*) in pubertal girls [40], suggesting that the decline in 25(OH)D levels during adolescence is expected and perhaps normal.

#### Obesity

In the current study, 24.6% and 18.6% of children were overweight and obese respectively, and a linear decline in 25(OH)D with higher BMI was found in both girls and boys. However, only in girls were 25(OH)D levels significantly associated with BMI and obesity index. Obesity is a common risk factor for low 25(OH)D globally [3, 29]. Measures that identify obesity may show different associations with 25(OH)D levels. In our previous study, BMI was not a significant predictor of 25(OH)D but waist/hip ratio was. Females with a > 0.75 WHR were twice as likely to have 25(OH)D < 25 nmol/L [9].

### Associations among health outcomes and low vitamin D in children

A number of health outcomes have been associated with vitamin D levels in the Middle East [3]. The most acknowledged outcomes in children are rickets and bone mineralization [7, 12]. Earlier observational and intervention studies showed that higher 25(OH)D levels were protective against rickets and bone mineral resorption and effective in treating rickets and increasing bone mass [1, 41].

Recent studies, on the other hand, have found new associations with other non-calcemic health outcomes. In a case-control study of 1274 children (<16 years), Bener et al. [42] found that having 25(OH)D levels <50 nmol/L was the main predictor of asthma in children (*n* = 671) and that it occurred in 68.1% of the asthmatic children compared to 36% of the controls.

In another case-control study, the impact of 25(OH)D levels <25 nmol/L on atopic dermatitis in Chinese children was found to be significant and occurred in 47.8% of the patients compared to 26.6% of the controls [18]. The study also found that elevated IgE was more common in the subjects with 25(OH)D levels <25 nmol/L compared to subjects with 25(OH)D levels of 25–49.9 nmol/L (43.2% vs 20%). However, a conflicting finding, with regard to dermatitis, was reported in a study on 1–17-year-old children in Germany for whom 25(OH)D levels were significantly higher in those with eczema compared to those without eczema [43].

Further, in the National Health and Nutrition Examination Survey in the US, 1–21 year old children had their hemoglobin and 25(OH)D levels evaluated. The results showed that those with 25(OH)D levels <75 nmol/L were almost twice as likely to be anemic (OR1.93, 95% CI: 1.21–3.08). Anemia was defined as a hemoglobin level < 5th percentile for age and sex [17].

A meta-analysis of 17 cross-sectional studies evaluating the relation between 25(OH)D levels and lipid profile in children and adolescence found a weak, but significant, inverse association between 25(OH)D levels and triglycerides and total cholesterol [44].

With regard to diabetes, serum 25(OH)D has been shown to be associated with type 1 diabetes mellitus in a number of studies in the Middle East. A study in Qatar found that having 25(OH)D levels <50 nmol/L was significantly more common in children with type 1 diabetes mellitus compared to healthy controls (28.8% vs 17%) and that mean serum levels of 25(OH)D were 38 ± 22.1 and 44.28 ± 22.9 respectively [16]. In a similar case-control study in Kuwait, Rasoul et al. [19] found that having 25(OH)D levels <50 nmol/L was associated with early onset of type 1 diabetes in children. This finding is alarming because 56.3% of the children in our current study had levels <50 nmol/L and that type 1 diabetes is highly prevalent in Kuwait (incidence rate 20.1 per 100,000 in 0–14 years children 95% CI 18–22.1) and is on the rise [45].

A possible link between type 1 diabetes and 25(OH)D has been suggested to be via its inverse correlation with HbA1c and its positive correlation with insulin, which is mediated by body fat [46]. Therefore, this finding may further suggest that obese children with low 25(OH)D have a greater risk for developing impaired glucose metabolism [46].

Without attempting to determine whether low 25(OH)D levels are a result or a cause at this stage, the association between 25(OH)D and a wide range of health problems suggests that low 25(OH)D levels may act as a marker for illness.

Our previous finding that 20% of the adolescent females (10–18 years) suffered from either osteopenia or osteoporosis was alarming, especially when coupled with 78.5% of the females having 25(OH)D levels <25 nmol/L [9]. In this current study, however, we found that 25(OH)D levels that begin to decrease before adolescence have a greater negative impact on bone mass and bone accrual.

Trying to raise 25(OH)D levels with vitamin D supplementation may not be protective against associated health issues [47]. Many studies, in fact, found that supplementing with vitamin D may have a negative impact [48, 49]. Clearly, serious measures to improve the health of children should be conducted, including improvements in diet and lifestyle.

## Limitations

There were certain limitations with regard to data collection in this study. Validated and standardized questionnaires to obtain data on physical activity and sun exposure were not used due to the limited time available for the child’s assessment. It is acknowledged that results could have been different had these questionnaires been used. Additionally, a bigger sample size would have improved our ability to examine the outcomes.

## Conclusions

Our results show relatively low levels of 25(OH)D in young schoolchildren (5–11 years old) in Kuwait, with significantly lower levels in girls. Given the association among 25(OH)D levels and a wide range of health outcomes, it is necessary to further examine the associated risk factors and implement major, holistic improvements in the lifestyles of these children.

## Abbreviations

25(OH)D: 25-hydroxyvitamin D
Adj-Ca: Adjusted serum calcium
BMI: Body mass index
IQR: Interquartile range
K-W: Kruskal-Wallis H test
M-W: Mann-Whitney U test
PTH: Parathyroid hormone
SPSS: Statistical package for the social sciences
WHR: Waist hip ratio
